# Maximum correentropy-based robust Square-root Cubature Kalman Filter for vehicular cooperative navigation

**DOI:** 10.1038/s41598-023-50377-w

**Published:** 2023-12-27

**Authors:** Wei Sun, Xiaotong Zhang, Wei Ding, Heming Zhang, Ao Liu

**Affiliations:** School of Geomatics, Liaoning Technical University, Fuxin, 12300 Liaoning China

**Keywords:** Mathematics and computing, Computer science, Engineering, Aerospace engineering

## Abstract

As the core method of cooperative navigation, relative positioning plays a key role in realizing intelligent vehicle driving and vehicle self-assembling network collaboration algorithms. However, when the contamination rate of measurement noise is high, the performance of filtering will be seriously affected. To better address the filtering performance degradation problem due to noise contamination, this paper proposes a vehicular cooperative localization method based on the Maximum Correentropy Robust Square-root Cubature Kalman Filter (MCSCKF). The algorithm not only retains the advantages of Square-root Cubature Kalman Filter (SCKF) but also has strong robustness to non-Gaussian noise. The experimental results of tightly integrated vehicular cooperative navigation show that compared with the Extended Kalman Filter (EKF) and Cubature Kalman Filter (CKF), the localization accuracy of MCSCKF is improved by 35.08% and 31.83%, respectively, which verified the effectiveness in improving the accuracy and robustness of the relative position estimation.

## Introduction

With the rapid development of intelligent transportation systems, vehicular positioning technology has become an important research field. Relative position sensing is not only the core of cooperative localization but also the key technology of intelligent vehicle driving^1^. Global Navigation Satellite System (GNSS) such as Global Positioning System (GPS) and BeiDou Navigation Satellite System (BDS) are becoming increasingly mature and have been successfully applied to vehicle positioning. However, due to the complexity of our surrounding built environment, road conditions, and vehicle traffic, the integration of multi-satellite navigation systems increases the accuracy error to a certain extent and affects positioning performance. To solve such problems, wireless networks have facilitated the development of cooperative positioning in Vehicular Ad Hoc Networks (VANETs)^2^.

Xu et al.^3^ proposed a method to obtain the relative position based on the Doppler shift, but it is infrastructure-dependent to be realized. Alam et al.^4^ proposed a Doppler-based cooperative positioning method for vehicular networks with GPS availability, proposing the use of Doppler shifts based on dedicated short-range communication (DSRC) signal carriers to improve GPS accuracy. The tight integration approach was proposed, taking into account this and emerging vehicular communication technologies, a method was proposed to improve relative positioning between two vehicles within a Vehicle Ad Hoc Network, fusing available low-level GPS data^5,6^. Feng Shen et al. proposed a new vehicular collaboration method, a novel tight cooperative positioning method based on the distance measurements of ultra-wideband (UWB), for relative positioning in new intelligent transportation systems. The method shares GPS pseudorange and Doppler shift measurements between participating vehicles, and then, each vehicle fuses GPS measurements and UWB-based distance to obtain relative position to avoid collision and improve driving safety^7^.

Although the accuracy of the relative distance estimation scheme for cooperative vehicle localization using a tight integration of GPS underlying data and UWB is improved, the data processing method used in the above technique is Extended Kalman Filtering (EKF). This method directly approximates the Gaussian integral with Taylor-expanded truncation, which can only achieve first-order accuracy and has a simple structure for systems with low nonlinearity. However, for tightly integrated GPS/UWB integration, the observation model has strong nonlinearities, since the EKF ignores higher-order terms, it will greatly reduce the filtering accuracy and may even cause divergence when the nonlinearity is high or the initial error is large. Therefore, the EKF needs to be improved and optimized.

To solve the nonlinear problems, the unscented Kalman filter (UKF) and the cubature Kalman Filter (CKF) are commonly used^8,9^. The unscented Kalman filter, first proposed by Juiler et al., was developed on the basis of the unscented transformation (UT)^10^. The elementary concept of UT is a method to compute the statistical properties of random variables that have been nonlinearly transformed, which approximates the a posteriori mean and variance of a nonlinear function by obtaining a set of sigma points through a certain sampling strategy and setting the corresponding mean weights and variance weights. The UKF approximates the statistical properties of the random quantity with a finite number of parameters, in other words, it conveys the statistical properties of the random quantity with a set of accurately chosen sampling points mapped by a nonlinear model, which fully reflect the true mean and covariance of the Gaussian density. The mean and covariance of the random quantities are then estimated by weighted statistical linear regression. The UKF does not introduce linearization error, thus it can reach the second-order accuracy of the Taylor series expansion, and there is no need to compute the Jacobian matrix. Therefore, it can be easily applied to the estimation of the state of a nonlinear system^11,12^. Driedger et al.^13^ found that EKF and UKF were applied to evaluate the feasibility of optical navigation based on resident space objects, and experiments showed that UKF was more reliable than EKF. Deori et al.^14^ used EKF and UKF to design and test the benchmark cart pendulum system and the underactuated offshore boom crane system, and the results showed that the accuracy of UKF was higher than that of EKF. However, some statistical properties of the Sigma points for the a posterior distribution of the nonlinear function are lost when the system dimension is higher, which can degrade the system estimation accuracy^15,16^.

In response to the above issues, Arasaratnam et al.^17^ proposed CKF, an algorithm based on the third-order spherical-radial cubature rule, which is based on the a priori mean and covariance. The Sigma cubature points are selected by the cubature rule, then these cubature points are passed through a nonlinear function, and then the cubature points after the nonlinear function are passed through are weighted to deal with the approximation of the state a posteriori mean and covariance. Because the filtering process of CKF also needs to conduct the decomposition and inverse of the error covariance matrix, it is necessary to ensure the positive characterization of the error covariance matrix. However, in practice, it is often difficult to ensure the positive definiteness. Thereby the Square-root Cubature Kalman Filter (SCKF) is proposed^18^, which directly updating the recursion in the form of the square root of the covariance matrix not only reduces the computational complexity and improves the efficiency, but also ensures the positive characterization of the covariance matrix and effectively avoids the divergence problem of the filter.

For the filtering problem under non-Gaussian conditions, many scholars have proposed a number of robust methods. Particle filtering (PF) and its improved algorithms use particles to deal with non-Gaussian noise issues^19,20^. These particle-based filtering methods use a large number of particles to approximate the probability distribution of the state and therefore also suffer from high computational complexity. Huber-based Kalman filtering is another popular method in recent years that uses a maximum likelihood regression criterion to deal with problems caused by non-Gaussian noise^21^, Tseng et al. applied their fusion with CKF to GPS navigation processing, and the results showed that the problem of contamination of measurements due to outliers or deviations from the assumption of Gaussian distribution, as well as the problem of contamination of signals by non-Gaussian noise or outliers, has been greatly improved^22^. Nevertheless, Huber-based methods usually select measurements that contain large errors, which may lead to non-negligible errors in the filter.

In the current state, the maximum correntropy criterion (MCC) has been introduced in the filter to deal with the problems caused by non-Gaussian noise^23,24^. The maximum correntropy Kalman filters^24^ are mainly applied to linear systems, and the maximum correntropy unscented Kalman filters^23,25^ are extended for solving some nonlinear problems. Nonetheless, they are not applicable to high-dimensional nonlinear systems. Therefore, this paper proposes a new nonlinear filter, Maximum Correntropy Square-root Cubature Kalman Filter (MCSCKF) based on the use of MCC to change the measurement update process of SCKF. This method not only has the advantages of SCKF as well as the overall line of thought, but also has strong robustness to non-Gaussian noise. Based on the work of Shen et al.^7^, experiments on vehicular cooperative navigation with a tight integration of GPS/UWB are conducted to further improve the performance of cooperative localization and enrich the research field for nonlinearities in practical applications of vehicle cooperative navigation. The experimental results show that MCSCKF is suitable for high-dimensional nonlinear systems, and the algorithm improves the estimation accuracy of the relative position.

The rest of the paper is organized as follows. Firstly, “Measurement and system modeling” introduces the measurement and system model for tight integration. Secondly, “Filtering algorithms” provides a preliminary introduction to MCC and SCKF. Then MCSCKF along with this algorithmic flow is derived in “MCSCKF algorithm”. After that, “Experimental analysis of on-board results” conducts on-board experiments and analyzes the results. Finally, “Conclusion” concludes the full paper with a summary and an outlook.

## Methods

### Measurement and system modeling

**Figure 1:**
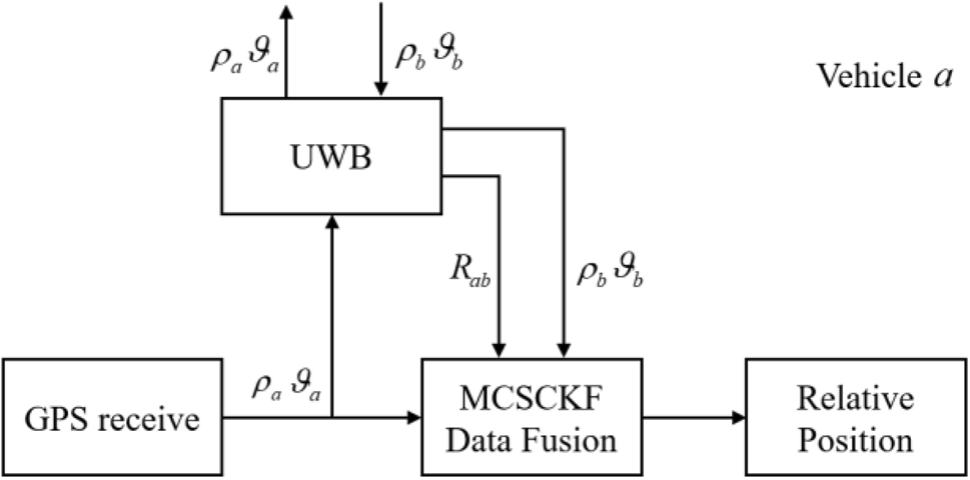
Relative sensor structure.

As shown in Fig. 1, only the relative motion of vehicle $a$ and vehicle $b$ in the vehicular ad hoc network is considered for convenience, where the GPS and UWB observations are fused in the process of tight-integration cooperative localization. Vehicle $a$ obtains the pseudo-range($\rho_{b}$) and Doppler shift($\vartheta_{b}$) of vehicle $b$ through UWB communication, then combines the local pseudo-range($\rho_{a}$) and Doppler shift ($\vartheta_{a}$)for double difference. At the same time, the UWB measures the distances of the two vehicles and carries out the data fusion process using MCSCKF to realize the relative localization of the vehicles.

#### GPS observations

The pseudorange between the receiver $a$ and the satellite $s$ at the moment $t$ is defined as the following equation:

1 $$\rho_{a}^{s} (t) = R_{a}^{s} (t) + \delta_{a} (t) + d^{s} + \zeta_{a}^{s} (t)$$

where $\rho_{a}^{s} (t)$ is the pseudorange between the receiver $a$ and the satellite $s$ at time $t$; $R_{a}^{s} (t)$ is the true distance between satellite $s$ and receiver $a$; $\delta_{a} (t)$ is the clock difference of receiver $a$; $d^{s}$ contains the satellite bias, atmospheric delay error, and other common errors of satellite $s$; and $\zeta_{a}^{s} (t)$ includes the thermal noise, multipath problem, and other non-disclosed systematic errors of receiver $a$ associated with satellite $s$.

When receivers $a$ and $b$ observe both satellite $s$ and satellite $j$, the pseudorange values derived from Eq. (1) can be eliminated by performing a pseudorange double-difference that eliminates the receiver's clock difference as well as other common satellite errors to obtain the following equation:

2 $$\rho_{ab}^{sj} (t) = R_{ab}^{sj} (t) + \zeta_{ab}^{sj} (t)$$

where $\rho_{ab}^{sj} (t)$ is the double differenced pseudorange between the receiver $a$ and receiver $b$ to two satellites $s$ and $j$, $R_{ab}^{sj} (t)$ is the double differenced geometric distance from two receivers to two satellites, $\zeta_{ab}^{sj} (t)$ is the error of receiver and satellite that can not be eliminated by the double difference. where $R_{ab}^{sj} (t)$ can be defined as:

3 $$R_{ab}^{sj} (t) = [\overset{\lower0.5em\hbox{$\smash{\scriptscriptstyle\rightharpoonup}$}}{{\mu_{s} }} (t) - \overset{\lower0.5em\hbox{$\smash{\scriptscriptstyle\rightharpoonup}$}}{{\mu_{j} }} (t)]^{T} \overset{\lower0.5em\hbox{$\smash{\scriptscriptstyle\rightharpoonup}$}}{{r_{ab} }} (t)$$

where $$\overset{\lower0.5em\hbox{$\smash{\scriptscriptstyle\rightharpoonup}$}}{{\mu_{s} }}$$ and $$\overset{\lower0.5em\hbox{$\smash{\scriptscriptstyle\rightharpoonup}$}}{{\mu_{j} }}$$ are the unit observation vectors from receiver $$a$$ (or receiver $$b$$) to the two satellites $$s$$ and $$j$$, respectively, and $$\overset{\lower0.5em\hbox{$\smash{\scriptscriptstyle\rightharpoonup}$}}{{r_{ab} }}$$ is the relative position vector of receiver $$a$$ and receiver $$b$$. Substituting Eq. (3) into Eq. (2) yields:

4 $$\rho_{ab}^{sj} (t) = [\overset{\lower0.5em\hbox{$\smash{\scriptscriptstyle\rightharpoonup}$}}{{\mu_{s} }} (t) - \overset{\lower0.5em\hbox{$\smash{\scriptscriptstyle\rightharpoonup}$}}{{\mu_{j} }} (t)]^{T} \overset{\lower0.5em\hbox{$\smash{\scriptscriptstyle\rightharpoonup}$}}{{r_{ab} }} (t) + \zeta_{ab}^{sj} (t)$$

Since the actual distance between the receiver and the satellite is about 20,000 km, and the GPS positioning error is negligible within a few tens of meters under these conditions, the apparent distance vector can be obtained by rough position estimation and a priori satellite ephemeris.

Similarly, the double differenced Doppler shift between receiver $a$ and receiver $b$ to satellite $s$ and satellite $j$ at the moment $t$ can be defined as:

5 $$\vartheta_{ab}^{sj} (t) = \frac{1}{\lambda }[\overset{\lower0.5em\hbox{$\smash{\scriptscriptstyle\rightharpoonup}$}}{{\mu_{s} }} (t) - \overset{\lower0.5em\hbox{$\smash{\scriptscriptstyle\rightharpoonup}$}}{{\mu_{j} }} (t)]^{T} \overset{\lower0.5em\hbox{$\smash{\scriptscriptstyle\rightharpoonup}$}}{{v_{ab} }} (t) + \gamma_{ab}^{sj} (t)$$

where $$\overset{\lower0.5em\hbox{$\smash{\scriptscriptstyle\rightharpoonup}$}}{{v_{ab} }}$$ is the relative velocity vector of receiver $$a$$ and receiver $$b$$, $$\lambda$$ is the wavelength of the GPS L1 signal, and $$\gamma_{ab}^{sj}$$ is the double differenced residual of the Doppler observation noise of receivers $$a$$ and $$b$$ with respect to satellite $$s$$ and satellite $$j$$.

#### UWB observations

UWB is a communication technology that uses narrow non-sinusoidal pulses of nanoseconds and microseconds to transmit data over short distances with a high transmission rate, low transmission power, and high penetration capability. The principle of its distance estimation is to estimate the distance by the signal propagation time between the base station and the target carrier, and to use the product of the arrival time of the UWB signal of the target carrier measured by the base station and the propagation speed as the relative distance between them. When using UWB for ranging, it is necessary to have measurement information from at least three base stations at the same time.

In the work of Shen et al.^7^, it is assumed that there is no non-line-of-sight (NLOS) problem for UWB between two vehicles, and the distribution law is an approximate Gaussian distribution function. To verify the specific performance of the MCSCKF, the actual measured distance across the UWB transceiver from two vehicles in 7 is used.

#### GPS/UWB tightly coupled system modeling

The system equation is defined as:

6 $$X(t + \tau ) = FX(t) + GD(t)$$

where $\tau$ is the observation period; $X$ is the state vector; $F$ is the state transition matrix, $G$ is the process noise model, and $D$ is the relative acceleration noise, which obeys a Gaussian distribution law with zero mean and standard deviation of $\sigma$ along each coordinate axis. Define the covariance matrix of the process noise as $Q = \sigma^{2} GG^{T}$, for receiver $a$ and receiver $b$, with the following equation:

7 $$X = [\begin{array}{*{20}c} {\overset{\lower0.5em\hbox{$\smash{\scriptscriptstyle\rightharpoonup}$}}{{r_{ab} }} } & {\overset{\lower0.5em\hbox{$\smash{\scriptscriptstyle\rightharpoonup}$}}{{v_{ab} }} } \\ \end{array} ]^{T} ,F = \left( {\begin{array}{*{20}c} {I_{3} } & {\tau I_{3} } \\ {0_{3} } & {I_{3} } \\ \end{array} } \right),G = [\begin{array}{*{20}c} {0.5\tau^{2} I_{3} } & {\tau I_{3} } \\ \end{array} ]^{T}$$

in which $I_{n}$ is an identity matrix of $n \times n$.

The observation equation for the relative localization of this experiment can be defined as:

8 $$y(t) = h(X(t)) + \zeta (t)$$

where $y$ is the observation vector, which includes the GPS pseudorange, the Doppler shift, and the actual distance between receivers $a$ and $b$ based on the UWB measurements; $h$ is a nonlinear function, which is derived from Eqs. (4) and (5) as well as the true relative distance between the two vehicles $\hat{R}_{ab} = \sqrt {\overrightarrow {{r_{ab} }}^{T} \overrightarrow {{r_{ab} }} }$; if the number of visible satellites of receivers $a$ and $b$ is $m + 1$, under this condition, the observation vector $y$ and the measurement noise $\zeta$ are expressed as follows:

9 $$y = [\begin{array}{*{20}c} {\rho_{ab}^{12} \cdots \rho_{ab}^{1m} } & {\vartheta_{ab}^{12} \cdots \vartheta_{ab}^{1m} } & {\zeta_{ab} } \\ \end{array} ]^{T}$$

10 $$\zeta = [\begin{array}{*{20}c} {\zeta_{ab}^{12} \cdots \zeta_{ab}^{1m} } & {\gamma_{ab}^{12} \cdots \gamma_{ab}^{1m} } & {\zeta_{ab} } \\ \end{array} ]^{T}$$

Assuming that the observations are independent of each other, the measurement noise covariance matrix can be defined as the following equation:

11 $$\Sigma = \left( {\begin{array}{*{20}c} {\Sigma_{c} } & {0_{m - 1} } & 0 \\ {0_{m - 1} } & {\Sigma_{\vartheta } } & 0 \\ {0_{1 \times (m - 1)} } & {0_{1 \times (m - 1)} } & {\Sigma_{r} } \\ \end{array} } \right)$$

If $\sigma_{\rho }^{2} ,\sigma_{\vartheta }^{2} ,\sigma_{r}^{2}$ are the variance of the pseudorange, the Doppler shift, and the UWB measurement error, respectively, it follows that.

12 $$\Sigma_{\rho } = \sigma_{\rho }^{2} AA^{T} ,\Sigma_{\vartheta } = \sigma_{\vartheta }^{2} AA^{T} ,\Sigma_{r} = \sigma_{r}^{2} AA^{T}$$

13 $$A = [\begin{array}{*{20}c} {1_{(m - 1) \times 1} } & { - I_{(m - 1)} } & { - 1_{(m - 1) \times 1} } & {I_{(m - 1)} } \\ \end{array} ]$$

where 1 is denoted as a matrix in which all elements are 1.

### Filtering algorithms

#### Maximum correntropy criterion

Given two random variables $X \in R,Y \in R$, assume that their joint distribution function is ${\text{F}}_{XY} (x,y)$; the entropy of correlation between the two is usually defined as:

14 $$V(X,Y) = {\text{E}} [\kappa (X,Y)] = \int {\kappa (x,y)d} {\text{F}}_{XY} (x,y)$$

where ${\text{E}} [ \cdot ]$ denotes the expected value and $\kappa ( \cdot , \cdot )$ is the Mercer kernel function, in this paper, we choose the Gaussian kernel as the kernel function of entropy, denoted as follows:

15 $$\kappa (x,y) = {\text{G}}_{\sigma } (e) = exp\left( { - \frac{{e^{2} }}{{2\sigma^{2} }}} \right)$$

here, $e = x - y$, $\sigma$ represent the kernel bandwidth while $\sigma$ > 0.

In practice, the joint distribution function ${\text{F}}_{XY} (x,y)$ is often unknown and the number of available data samples is limited. Therefore, we often use the sample mean estimator to estimate the correlation coefficient:

16 $$\hat{V}(X,Y) = \frac{1}{N}\sum\limits_{i = 1}^{N} {{\text{G}}_{\sigma } (e(i))}$$

where $e(i) = x(i) - y(i)$, and $\{ x(i),y(i)\}_{i = 1}^{N}$, denote the *N* sample data drawn from the joint distribution function ${\text{F}}_{XY} (x,y)$.

Using the entropy value as a cost function has a strong suppression effect on non-Gaussian noise. If a column of error data $\{ e(i)\}_{i = 1}^{N}$ is obtained, the MCC-based objective function is denoted as:

17 $$J_{MCC} = \frac{1}{N}\sum\limits_{i = 1}^{N} {{\text{G}}_{\sigma } (e(i))}$$

#### Square-root Cubature Kalman Filter

Square-root Cubature Kalman Filter (SCKF) is an effective method to solve the state estimation problem of nonlinear systems by transferring the square root form of the error covariance. In this paper, it can not only overcome the problem of low accuracy of EKF due to linearization but also avoid the problem of CKF error covariance losing positive characterization.

Taking the nonlinear system in Eqs. (6) and (8) as an example, the steps of time update and measurement update for SCKF are shown below:

##### Time update

Suppose that at time $k$, $S_{k - 1|k - 1}$ is the square root of the covariance matrix $P_{k - 1|k - 1}$, i.e., $P_{k - 1|k - 1} = S_{k - 1|k - 1} S^{T}_{k - 1|k - 1}$. Similarly, $S_{Q,k - 1} ,S_{R,k}$ are square root factors of $Q_{k - 1} ,R_{k}$, i.e., $Q_{k - 1} = S_{Q,k - 1} S^{T}_{Q,k - 1} ,R_{k} = S_{R,k} S^{T}_{R,k}$, respectively.

Evaluate the cubature points:

18 $$\chi_{i,k - 1|k - 1} = S_{k - 1|k - 1} \xi_{i} + \hat{x}_{k - 1|k - 1} ,i = 1,...,2n$$

where

19 $$\xi_{i} = \left\{ \begin{gathered} \sqrt n \left[ 1 \right]_{i} ,i = 1,...n \\ - \sqrt n \left[ 1 \right]_{i - n} ,i = n + 1,...2n \\ \end{gathered} \right.$$

here, $\xi$ denotes the $n \times n$-unit matrix, $\left[ 1 \right]_{i}$ denotes the $i{\text{ - th}}$ column vector.

Dissemination of cubature points:

20 $$\chi_{i,k|k - 1}^{ * } = f\left( {\chi_{i,k - 1|k - 1} } \right),i = 1,...,2n$$

Estimate the square root of the a prior state and the corresponding covariance matrix:

21 $$\hat{x}_{k|k - 1} = \frac{1}{2n}\sum\limits_{i = 1}^{2n} {\chi_{i,k|k - 1}^{ * } }$$

22 $$S_{k|k - 1} = Tria\left( {\left[ {{\rm X}_{k|k - 1}^{ * } ,S_{Q,k - 1} } \right]} \right)$$

where $Tria\left( \cdot \right)$ denotes the QR decomposition of the matrix, ${\rm X}_{k|k - 1}^{ * }$ from (23):

23 $${\rm X}_{k|k - 1}^{ * } = \frac{1}{{\sqrt {2n} }}\left[ {\chi_{1,k|k - 1}^{ * } - \hat{x}_{k|k - 1} ,...,\chi_{2n,k|k - 1}^{ * } - \hat{x}_{k|k - 1} } \right],i = 1,...,2n$$

##### Measurement update

Evaluate the cubature points:

24 $$\chi_{i,k|k - 1} = S_{k|k - 1} \xi_{i} + \hat{x}_{k|k - 1} ,i = 1,...,2n$$

Dissemination of cubature points:

25 $$\chi_{i,k|k - 1}^{ * * } = h\left( {\chi_{i,k|k - 1} } \right),i = 1,...,2n$$

Estimate the square root of the a priori measurements and the corresponding covariance matrix:

26 $$\hat{y}_{k|k - 1} = \frac{1}{2n}\sum\limits_{i = 1}^{2n} {\chi_{i,k|k - 1}^{ * * } }$$

27 $$S_{yy,k|k - 1} = Tria\left( {\left[ {{\text{Y}}_{k|k - 1} ,S_{R,k} } \right]} \right)$$

here ${\text{Y}}_{k|k - 1}$ is denoted as shown below:

28 $${\text{Y}}_{k|k - 1} = \frac{1}{{\sqrt {2n} }}\left[ {\chi_{1,k|k - 1}^{ * * } - \hat{y}_{k|k - 1} ,...,\chi_{2n,k|k - 1}^{ * * } - \hat{y}_{k|k - 1} } \right],i = 1,...,2n$$

Calculate the inter-correlation covariance matrix:

29 $$S_{xy,k|k - 1} = {\rm X}_{k|k - 1} {\text{Y}}_{k|k - 1}^{T}$$

with ${\rm X}_{k|k - 1}$ in the equation:

30 $${\rm X}_{k|k - 1} = \frac{1}{{\sqrt {2n} }}\left[ {\chi_{1,k|k - 1} - \hat{x}_{k|k - 1} ,...,\chi_{2n,k|k - 1} - \hat{x}_{k|k - 1} } \right],i = 1,...,2n$$

Calculate the Kalman gain:

31 $${\rm K}_{k} = {{\left( {{{S_{xy,k|k - 1} } \mathord{\left/ {\vphantom {{S_{xy,k|k - 1} } {S_{yy,k|k - 1}^{T} }}} \right. \kern-0pt} {S_{yy,k|k - 1}^{T} }}} \right)} \mathord{\left/ {\vphantom {{\left( {{{S_{xy,k|k - 1} } \mathord{\left/ {\vphantom {{S_{xy,k|k - 1} } {S_{yy,k|k - 1}^{T} }}} \right. \kern-0pt} {S_{yy,k|k - 1}^{T} }}} \right)} {S_{yy,k|k - 1} }}} \right. \kern-0pt} {S_{yy,k|k - 1} }}$$

Estimate the square root of the a posterior state and the a posterior covariance matrix:

32 $$\hat{x}_{k|k} = \hat{x}_{k|k - 1} + {\rm K}_{k} \left( {y_{k} - \hat{y}_{k|k - 1} } \right)$$

33 $$S_{k|k} = Tria\left( {\left[ {{\rm X}_{k|k - 1} - {\rm K}_{k} {\text{Y}}_{k|k - 1} ,{\rm K}_{k} S_{R,k} } \right]} \right)$$

### MCSCKF algorithm

Due to the excellent performance of correntropy in non-Gaussian noise environments^26^, we combine MCC with SCKF and use MCC to improve the robustness of SCKF.

First, from the nonlinear model described by Eqs. (6) and (8), the a priori estimated states and the corresponding square root covariance matrices are evaluated by Eqs. (18)–(23).

Then, the nonlinear regression model is constructed by combining Eqs. (8), (21), and (22) as follows.

34 $$\left[ \begin{gathered} \hat{x}_{k|k - 1} \\ y_{k} \\ \end{gathered} \right] = \left[ \begin{gathered} x_{k} \\ h\left( {x_{k} } \right) \\ \end{gathered} \right] + \varphi_{k}$$

where $\varphi_{k}$ = $$\left[ \begin{gathered} \hat{x}_{k|k - 1} - x_{k} \\ \zeta_{k} \\ \end{gathered} \right]$$, given by the square root of the covariance matrix $\varphi_{k}$:

35 $$B_{k} = \left[ {\begin{array}{*{20}c} {S_{k|k - 1} } & 0 \\ 0 & {S_{R,k} } \\ \end{array} } \right]$$

Equation (34) is obtained by multiplying both sides of $B_{k}^{ - 1}$ simultaneously:

36 $$D_{k} = g\left( {x_{k} } \right) + e_{k}$$

the specific expression in the above equation is as shown below:

37 $$D_{k} = B_{k}^{ - 1} \left[ \begin{gathered} \hat{x}_{k|k - 1} \\ y_{k} \\ \end{gathered} \right],g\left( {x_{k} } \right) = B_{k}^{ - 1} \left[ \begin{gathered} x_{k} \\ h\left( {x_{k} } \right) \\ \end{gathered} \right],e_{k} = B_{k}^{ - 1} \varphi_{k}$$

Based on the above model, the cost function MCC is constructed as:

38 $$J_{MCC} \left( {x_{k} } \right) = \sum\limits_{i = 1}^{n + m} {G_{\sigma } \left( {e_{i,k} } \right)}$$

where $e_{i,k} = d_{i,k} - g_{i,k}$, and here $d_{i,k} ,g_{i,k}$ are the $i - th$ component of $D_{k} ,g\left( {x_{k} } \right)$, respectively.

Then the $x_{k}$ optimal estimate based on MCC can be obtained from the following equation:

39 $$\hat{x}_{k} = \mathop {\arg \max }\limits_{{x_{k} }} \sum\limits_{i = 1}^{n + m} {G_{\sigma } \left( {e_{i,k} } \right)}$$

Let the first order derivative of the cost function is equal to zero, then we can derive:

40 $$\sum\limits_{i = 1}^{n + m} {\psi \left( {e_{i,k} } \right)} \frac{{\partial e_{i,k} }}{{\partial x_{k} }} = 0$$

here, $$\psi \left( {e_{i,k} } \right) = G_{\sigma } \left( {e_{i,k} } \right) \cdot e_{i,k}$$.

Then, we define $C_{i,k} = {{\psi \left( {e_{i,k} } \right)} \mathord{\left/ {\vphantom {{\psi \left( {e_{i,k} } \right)} {e_{i,k} = }}} \right. \kern-0pt} {e_{i,k} = }}G_{\sigma } \left( {e_{i,k} } \right)$, and there it is:

41 $$C_{k} = diag\left( {C_{1,k} ,...,C_{n + m,k} } \right) = \left[ {\begin{array}{*{20}c} {C_{x,k} } & 0 \\ 0 & {C_{y,k} } \\ \end{array} } \right]$$

where $$C_{x,k} = diag\left( {C_{1,k} ,...,C_{n,k} } \right),C_{y,k} = diag\left( {C_{1,k} ,...,C_{m,k} } \right)$$.

According to (41), (40) can further be denoted as:

42 $$\left( {\frac{{\partial g\left( {x_{k} } \right)}}{{\partial x_{k} }}} \right)^{T} C_{k} \left( {D_{k} - g\left( {x_{k} } \right)} \right) = 0$$

In fact, the key to improving SCKF performance using MCC is to use $C_{k}$ to update the state covariance and the variance of the measurement noise.

The definition of $\Phi_{k}$ is the updated covariance matrix, denoted as follows:

43 $$\Phi_{k} = B_{k} C_{k}^{ - 1} B_{k}^{T}$$

For the next derivation, we write $\Phi_{k}$ in block matrix form, such that:

44 $$\Phi_{k} = \left[ {\begin{array}{*{20}c} {\Phi_{x,k} } & 0 \\ 0 & {\Phi_{y,k} } \\ \end{array} } \right]$$

in fact, we can derive

45 $$\Phi_{x,k} = S_{k|k - 1} \cdot {\rm I} \cdot S_{k|k - 1}^{T} = P_{k|k - 1}$$

the updated measurement covariance matrix is also derived:

46 $$R_{k}^{ * } = \Phi_{y,k}$$

which is then the square root of the updated measurement covariance matrix:

47 $$R_{k}^{ * } = S_{R,k}^{ * } S_{R,k}^{ * T}$$

The main steps of the MCSCKF algorithm are summarized as follows.

(1) Choose a suitable kernel bandwidth $\sigma$, assume an initial estimation state of $\hat{x}_{0|0}$ and an associated square root covariance matrix ${\text{S}}_{0|0}$, and set the time $k = 1$;

(2) In the same time update step as the Square-root Cubature Kalman Filter (SCKF), the MCSCKF performs calculations (18)–(23);

(3) Derive the updated square root covariance matrix of measurements $S_{R,k}^{ * }$ from (34)–(47), compute the a priori measurement means using (26), replace $S_{R,k}$ with $S_{R,k}^{ * }$ from (27), and thus obtain the corresponding updated square root covariance matrix:

48 $$S_{yy,k|k - 1} = Tria\left( {\left[ {{\text{Y}}_{k|k - 1} ,S_{R,k}^{ * } } \right]} \right)$$

(4) Perform the MCSCKF measurement update process using (26), (48), and (29) through (32) and (49), and then return to (2) for the next time update when $k = k + 1$ occurs.

49 $$S_{k|k} = Tria\left( {\left[ {{\rm X}_{k|k - 1} - {\rm K}_{k} {\text{Y}}_{k|k - 1} ,{\rm K}_{k} S_{R,k}^{ * } } \right]} \right)$$

It is worth noting that the kernel bandwidth $\sigma$ in step 1) is a key parameter in the MCSCKF algorithm, larger or smaller kernel widths cannot optimize the performance of the algorithm^27^. The smaller the kernel bandwidth, the more relatively stable and robust the algorithm turns out to be. In case the kernel bandwidth is much too narrow. However, the filtering accuracy will be reduced, slowing down the convergence of the filtering or even leading to filtering divergence. On the other hand, when the kernel bandwidth is too large, MCSCKF degenerates to SCKF^28^. Therefore, the value of $\sigma$ should be chosen appropriately, and after many experiments and summarized by previous authors, the value of $\sigma$ is 3 in this paper.

### Ethics declarations

This research project has been approved by the relevant ethical committee or institution and was conducted in strict accordance with ethical guidelines. In this study, we respected and protected the rights and privacy of the participants and ensured the confidentiality of their personal information.

### Participant informed consent

We explained the purpose, process, risks, and benefits of the study to all individuals participating in the study either verbally or in writing and obtained their informed consent. Participants had the right to know that their participation was voluntary and that they could withdraw from the study at any time.

### Data confidentiality and privacy protection

We have taken appropriate measures to protect the privacy of participants' personal information. We will not publicize or disclose any personal information that could lead to the identification of participants. We will anonymize participant information in research reports. This statement is intended to safeguard the ethical compliance of the research project and to protect the rights and privacy of the participants. Please feel free to contact us with any further questions or concerns.

## Results

### Experimental analysis of on-board results

In order to verify the adaptability and robustness of MCSCKF together with a comparative analysis of the effects of various filters on the performance of cooperative navigation, the experimental data used in this experiment are the same as those in the literature^7^. Two vehicles were driven at different speeds, relative speeds, and distances. The maximum speed was 80 km/h, the maximum relative speed was 34 km/h, and the maximum distance between them was 78 m. A set of low-cost Xsens MTI-G INS is installed on two vehicles, $a$ and $b$, to obtain the raw data needed by the algorithm, in addition to simultaneously obtaining the actual distances of the two vehicles via MSSI-based UWB. At the same time, a Leica GS10 receiver and a set of Novatel IMU-LCI integrated GNSS carrier phase differences are equipped on both vehicles for high-precision position estimation to get the reference solutions, with a data sampling rate of 1 Hz and an experimental duration of thirteen minutes.

**Figure 2:**
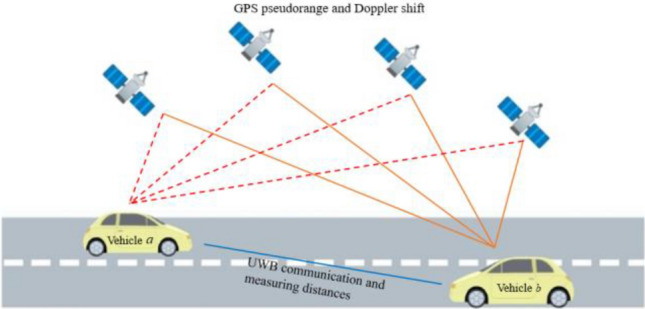
Schematic of the experimental settings.

During the experiment, both vehicles are in motion. The GPS receiver on the carrier is used to observe the visible satellites in the experimental area in real-time, to get the pseudorange and Doppler shift between the vehicles and the satellites, and to obtain the real-time distance between the two vehicles as well as the observation information through UWB. To ensure time synchronization, the GPS time is used for data synchronization. The algorithm of this experiment utilizes the L1 pseudorange, the Doppler shift, and the real distance between the two vehicles based on UWB as the observation data to establish the data fusion model as shown in Fig. 2. EKF, classical CKF, and improved MCSCKF are compared in this experiment to analyze the data fusion and draw conclusions.

**Figure 3:**
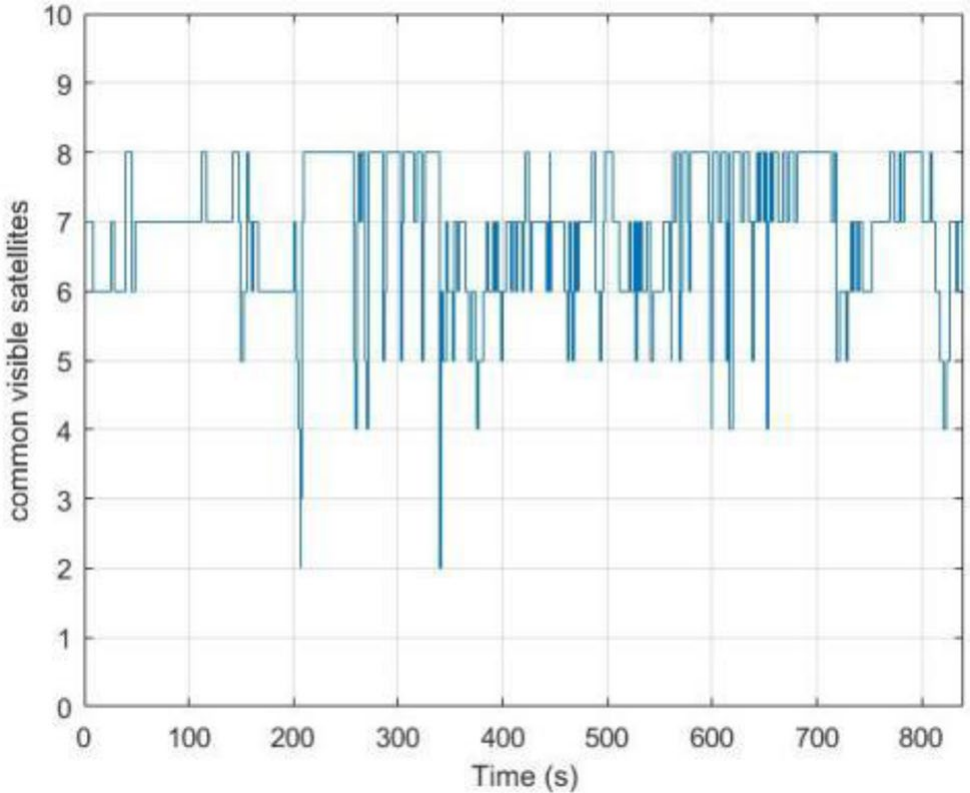
Number of commonly visible satellites.

**Figure 4:**
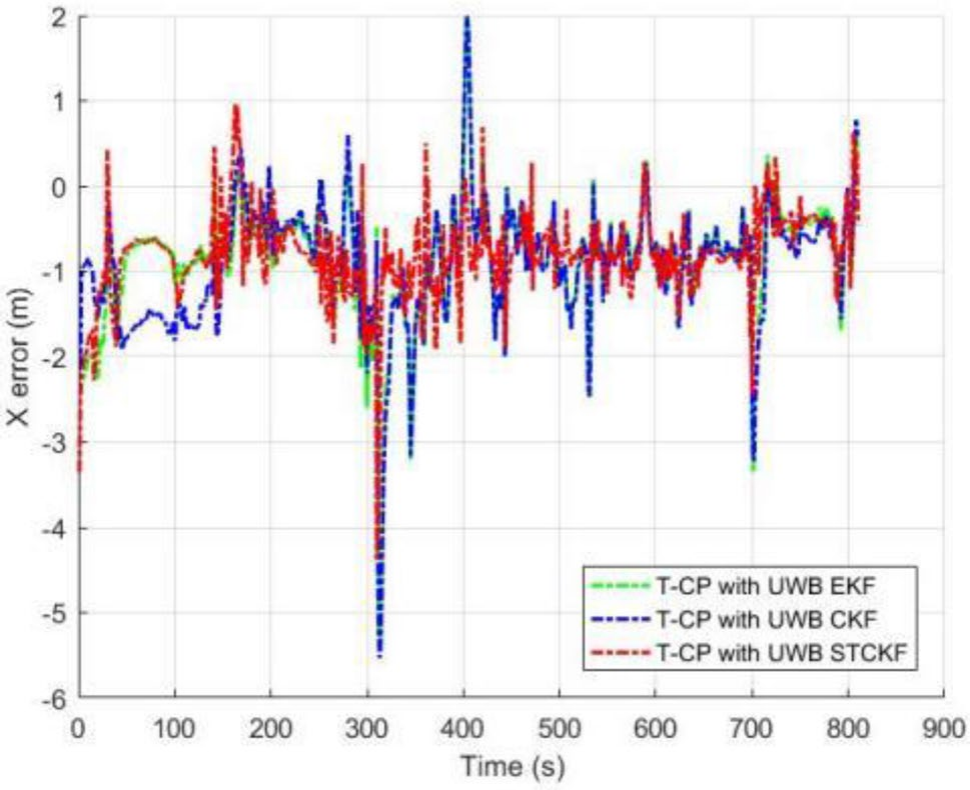
Comparison of X-axis errors.

**Figure 5:**
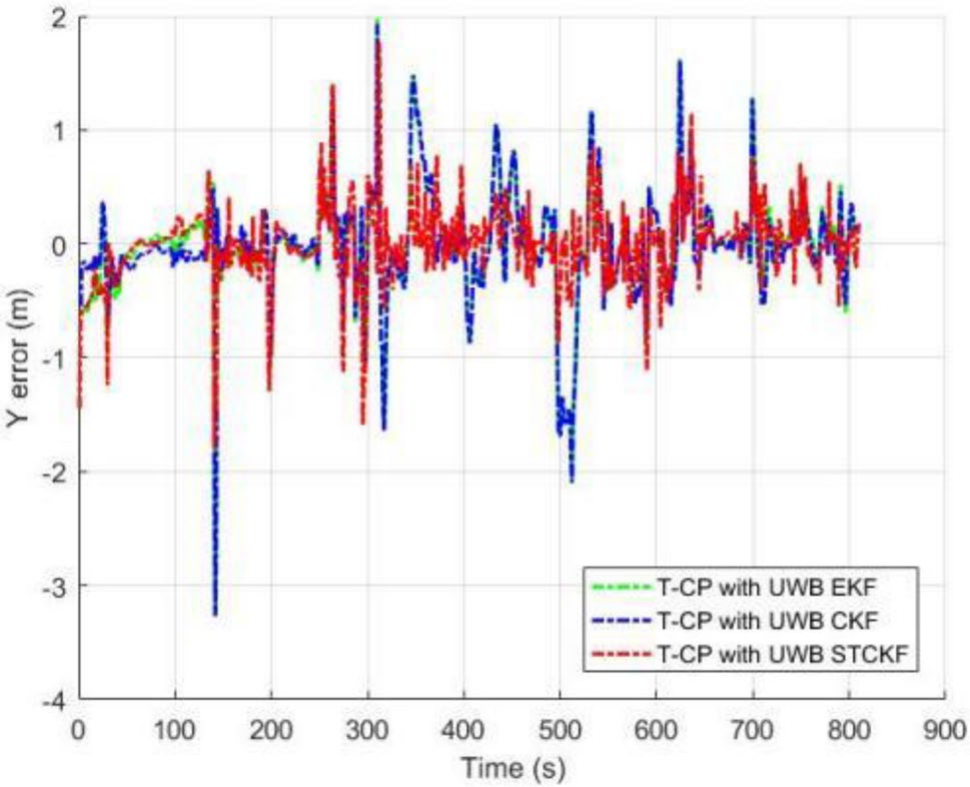
Comparison of Y-axis errors.

**Figure 6:**
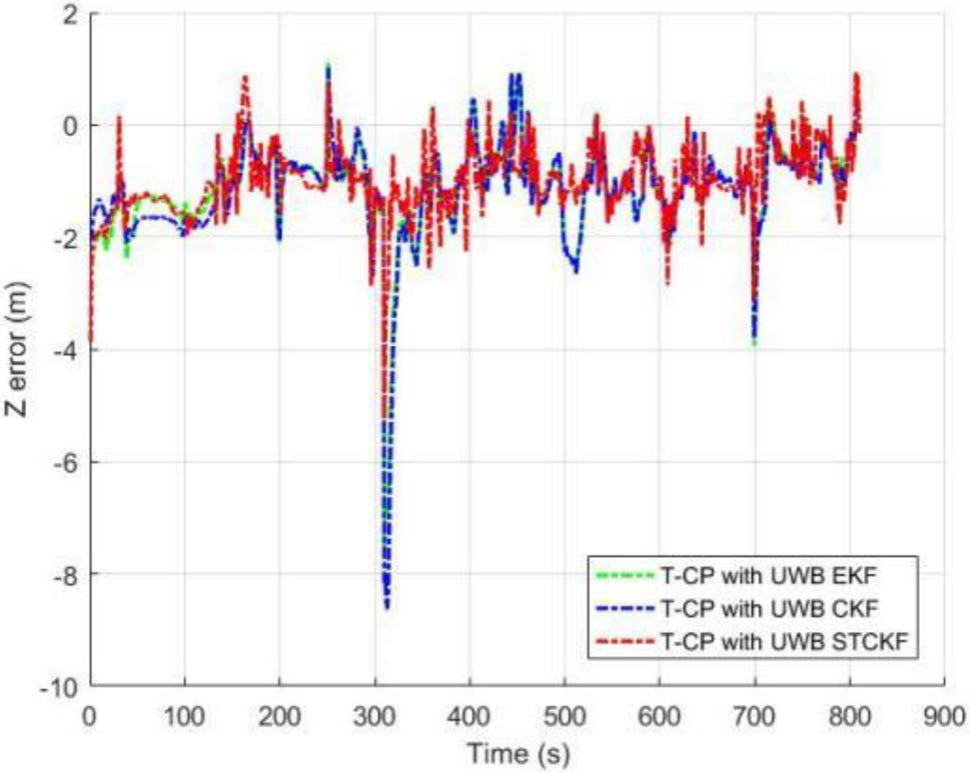
Comparison of Z-axis errors.

**Figure 7:**
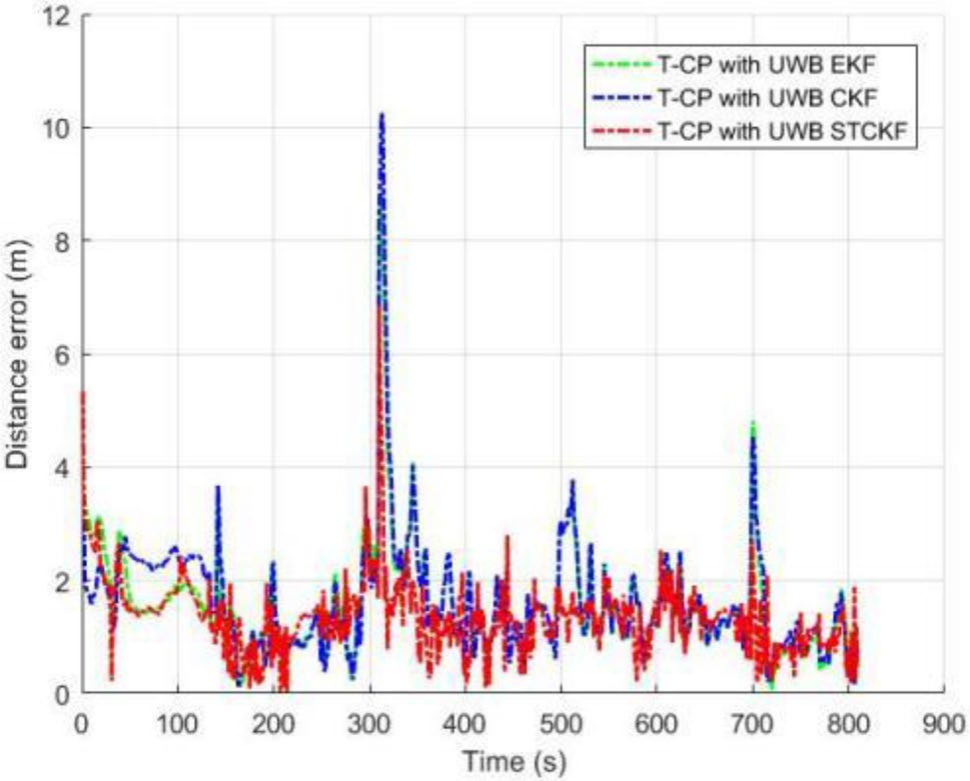
Comparison of three-axis distance errors.

As shown in Fig. 3, the number of visible satellites needs to be greater than 4 satellites in all other common experiments. In the occasional case of less than 4 satellites, this leads to a decrease in the Kalman filtered innovation and the covariance matrix dimension of the observations, which results in the measurement update not being performed properly, and in order to ensure that the measurement update is performed properly, the Kalman filter can compensate for this by using the dynamic models of the system, setting the innovation of missing observation to zero, and the observation covariance matrix is changed to infinity as a consequence. Figures 4, 5, 6 and 7 show the resulting three-axis error as well as the overall error, respectively.

**Figure 8:**
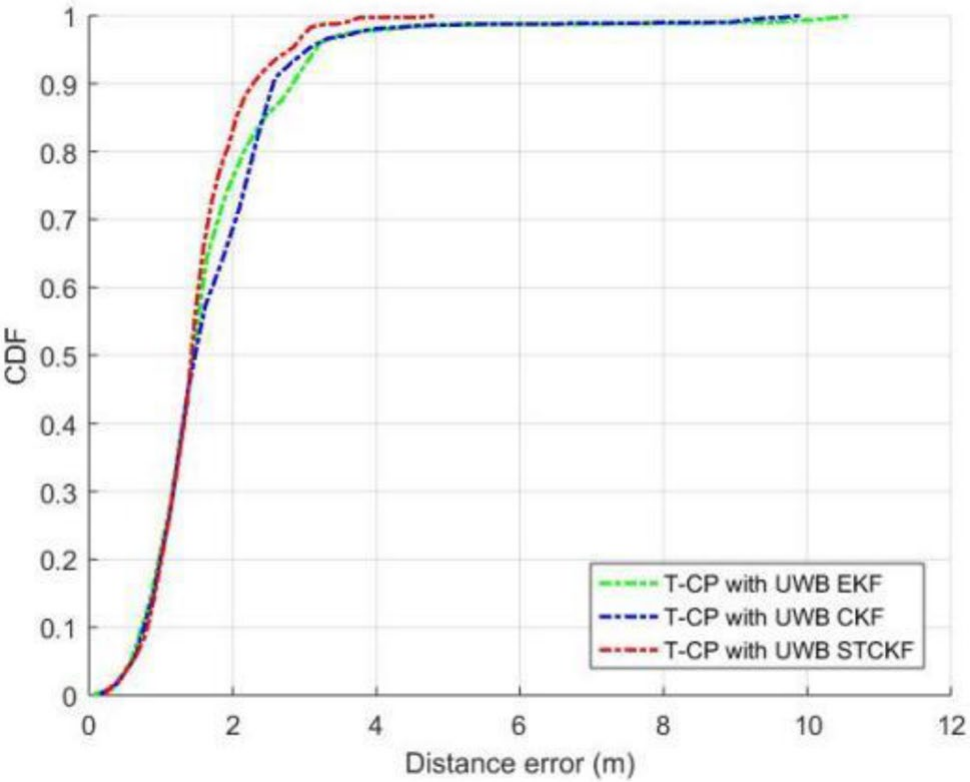
Comparison of error cumulative distribution functions.

As shown in Fig. 7, EKF and CKF have basically the same position estimation accuracy, while the positioning accuracy of MCSCKF is significantly better than the two, which utilizes a nonlinear system to transmit the cubature law, and at the same time integrates the rules of Maximum Correntropy Criterion and Square-root Cubature Kalman Filter to inhibit the influence of colored noise in the actual measurements on the experimental data, and accordingly, it can be concluded that the precision, accuracy and robustness are better than EKF and CKF. Figure 8 represents the error cumulative distribution function (CDF) of the three different filters in the experiment, which shows that the applicability of MCSCKF is significantly better than that of the other two Kalman filters.

On this basis, in order to better analyze the effect of different filters on the position estimation accuracy of the experiments, the quality of the estimation is expressed using the Root Mean Square Error (RMSE). For further analysis, the accuracy ($e_{a}$) and precision ($e_{p}$) of the relative position estimation are also defined.

The quantitative results of the three can be expressed using the following equation:

50 $$e_{a} = \left| {m^{ - 1} \sum\limits_{t = 1}^{m} {[\overset{\lower0.5em\hbox{$\smash{\scriptscriptstyle\rightharpoonup}$}}{{r^{\prime}}} (t) - \mathop{r}\limits^{\rightharpoonup} (t)]} } \right|$$

51 $$e_{p} = \sqrt {\sum\limits_{i = 1}^{3} {\Omega_{ii} } }$$

where $$\overset{\lower0.5em\hbox{$\smash{\scriptscriptstyle\rightharpoonup}$}}{{r^{\prime}}}$$ and $$\mathop{r}\limits^{\rightharpoonup}$$ are the relative position estimated by the algorithm and the reference relative position of the RTK, respectively, $$m$$ is the total number of calendar elements, $$\Omega_{ii}$$ is the ith value in the diagonal of the $$\Omega$$ matrix, with $$\Omega = {\text{cov}} [\overset{\lower0.5em\hbox{$\smash{\scriptscriptstyle\rightharpoonup}$}}{{r^{\prime}}} (t) - \mathop{r}\limits^{\rightharpoonup} (t)]$$. The performance metrics of the three filtering algorithms are shown in the following Table 1.

**Table 1:** Quantitative indicators of experimental results.

Method	RMS/m	Accuracy/m	Precision/m
EKF	2.05	1.52	1.38
CKF	2.03	1.55	1.31
MCSCKF	1.62	1.35	0.89

Define the parameter $\omega$ to be used to indicate the enhancement effect of scheme B on the realization of scheme A, where ErrorA and ErrorB are the values of the error metrics for the three performances in Table 2.

52 $$\omega = \left[ {1 - \frac{ErrorB}{{ErrorA}}} \right] \times 100\%$$

**Table 2:** Experimental results and performance improvement.

Method	RMS/m	Accuracy/m	Precision/m
MCSCKF over EKF	20.74%	10.64%	35.08%
MCSCKF over CKF	20.08%	12.64%	31.83%

Using the above equation, the percentage increase of MCSCKF over the other two classical filtering algorithms among the three performances is calculated as shown in Table 2:

Based on the above data, experimental results can be derived, which show that the relative position perception of MCSCKF has stronger robustness and localization accuracy than EKF and CKF, and also provides a more accurate system control method for tight combination cooperative navigation.

## Conclusion

Based on the theory of robustness and adaptability, the data processing method of vehicle relative position localization is optimized, which further improves the vehicle positioning accuracy and the performance of cooperative navigation. Moreover, in response to the problem that the covariance matrix dimension of the Kalman filter is reduced due to the new information and observations of the Kalman filter, which leads to the failure of the measurement update, a Maximum Correentropy-based Robust Square-root Cubature Kalman Filter is proposed, which improves the data fusion method, and further improves the accuracy of vehicle relative positioning, and also improves the performance of cooperative navigation. further improves the accuracy of relative vehicle localization, and also opens up a new field for cooperative navigation at the same time. The in-vehicle navigation experiments yielded that the positioning accuracy and robustness of MCSCKF improved by 20.74% and 35.08% compared with EKF; the positioning accuracy and robustness of MCSCKF improved by 20.08% and 31.83% compared with CKF, thus verifying the robustness and superiority of MCSCKF. However, the algorithm proposed in this paper also has a number of limiting factors, which depend on the actual situation problems in engineering, and these problems also limit the method to have limitations in complex applications in practice. This point is also the subject of subsequent research work.
